# Declining snowfall fraction in the alpine regions, Central Asia

**DOI:** 10.1038/s41598-020-60303-z

**Published:** 2020-02-26

**Authors:** Zhi Li, Yaning Chen, Yupeng Li, Yang Wang

**Affiliations:** 1 State Key Laboratory of Desert and Oasis Ecology, Xinjiang Institute of Ecology and Geography, Chinese Academy of Sciences, Urumqi, 830011 China; 2 University of Chinese Academy of Sciences, Beijing, 100049 China; 3 College of Pratacultural and Environmental Sciences, Xinjiang Agricultural University, Urumqi, 830052 China

**Keywords:** Climate-change mitigation, Hydrology

## Abstract

In a warming climate, precipitation (P) is less likely to occur as snowfall (S). Change in the snowfall fraction (S/P) is currently assumed not only influences the accumulation and ablation of glaciers, but also influences the streamflow and water resources significantly in mountainous regions. However, until now, most studies have focused on precipitation magnitude and its frequency changes, while seasonal shifts in precipitation types have been mostly neglected. This paper employs the threshold temperature method in combination with multi-source dataset (APHRODITE, CPC and meteorological stations) analysis to determine snowfall proportions in precipitation in the Tienshan Mountains, Central Asia, during 1960–2017. The results indicated that temperature-induced precipitation shifting from snow to rain. The S/P experienced an overall declining trend, increasing at a rate of 0.6%/decade prior to the mid-1990s, followed by a downward trend at a rate of −0.5%/decade. The S/P decreased mainly at low and middle altitudes (between 1500 and 3500 m). At higher altitudes (over 3500 m), the magnitudes of the decreased S/P ratios were small or even increased due to the temperature always being below freezing. Decreases in S/P are always associated with decreases in annual streamflow in the glacier/snow melt recharged rivers.

## Introduction

Precipitation over alpine regions can occur in different phases of liquid and solid. Snowfall, as the most important component of solid precipitation, is a key part of hydrological processes in the mountains during the cold season. It is also regarded as an indicator that reflects climatic change due to its high sensitivity to alterations in temperature and precipitation^1,2^. The snowfall fraction (S/P) is the ratio of snowfall (S) to precipitation (P), with changes in the S/P depending on alterations in snow and precipitation amounts^3,4^. As there may exist inconsistent changes in snowfall and precipitation, the snowfall fraction can be used as another effective indicator to reflect climate change^5^.

Most of the previous studies use temperature or wet-bulb temperature and relative humidity to define thresholds which demarcate the solid and liquid types of precipitation^6–8^. As global temperatures continue to warm, many regions experienced a decreased S/P. The S/P has shown a significant decreasing trend across the New England states^9^, Switzerland^5^, the continental United States^10,11^, the Tibetan Plateau^8,12^, and the Chinese Tienshan Mountains^3^. The S/P has a strong influence on mountainous hydrological processes. The performance of water resource management systems is highly related to changes in the S/P in the regions where the land surface hydrological process is dominated by snow accumulation in winter and melt in spring^6^. Decreases in the S/P can lead to increases in water loss during winter or early spring, whereas increases in the S/P indicate that much more water is being stored as snows until the temperature exceeds the melt point^4,13^. Two widely anticipated changes in the hydrological cycle are the warming-induced precipitation shifts from snow to rain and the earlier melt of the snowpack^14,15^. Both of these changes are associated with a significant impact on water resource utilization. Even without any changes in precipitation intensity, alterations in patterns of precipitation will result in a shift in peak river runoff^13^. However, until now, most studies have focused on precipitation magnitude and its frequency changes, while seasonal shifts in precipitation types have been mostly neglected.

**Figure 1:**
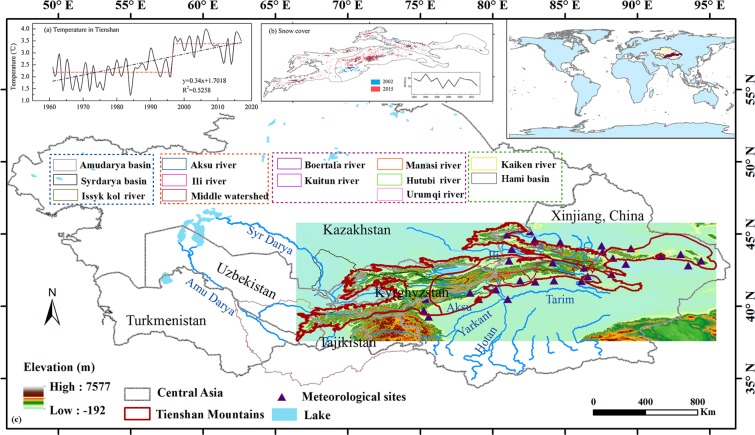
Sketch map of the Tienshan Mountains, Central Asia. (**a**) Temperature in Tienshan in the figure caption, (**b**) Maximum snow cover changes based on the MOD10A1 product, (**c**) river basins in the Tienshan Mountains, Central Asia (Generated by ArcGIS 10.3, URL: http://www.esri.com/software/arcgis/arcgis-for-desktop, and Matlab R2013a, URL: http://cn.mathworks.com/products/matlab/).

The Tienshan Mountains span from Uzbekistan to Kyrgyzstan, and south-eastern Kazakhstan to the Xinjiang, China, which cover a large portion of arid Central Asia (Fig. 1). The mountain range is an important component of the “Silk Road Economic Belt”. Water resources in arid Central Asia are mainly formed in the mountain areas because precipitation predominantly occurs in the mountains. This water source, together with water from glacier shrinkage and snowmelt, plays a vital role in maintaining the fragile ecosystem. With its important status as “water tower of Central Asia”^16^, the Tienshan Mountains is important to Central Asia’s natural environment and oasis security in arid areas^17^. Large range in elevation and various land surface characteristics in the Tienshan Mountains result in a great difference in spatiotemporal distribution of precipitation and snowfall across different temperature zones^18^. The Tienshan Mountains span several countries in Central Asia, creating a decentralized political entity of complex multi-national and multi-ethnic forms. These characteristics restrict the integrated research on this mountain range, so information in its entirety remains scarce.

Global warming is commonly reported in recent years, especially obvious in arid/semi-arid regions in the mid-latitude Northern Hemisphere^19^. The Tienshan Mountains are located in the Eurasian hinterland, where the increasing rate of average annual temperature has been around 0.34 °C/decade over 1960–2017 (Fig. 1a). The warming rate is significantly higher than the average global warming rate during the same period^15^. Typically, especially in cold seasons, temperature increases are negatively correlated with snow cover^20–22^. However, little is known about how a warming climate might shift forms of precipitation.

Given the high variability in sharply increased temperatures, how do spatio-temporal changes in the S/P and forms of precipitation in the Tienshan Mountains interact with differences in elevation as a consequence of ongoing global warming? The answer to this question is crucial for understanding mountainous runoff processes and the stability of the water system. However, due to the sparsity of the hydrometeorological station network in the highlands and observational data deficiency following the collapse of the Soviet Union, there is a critical shortage of analysis of snowfall fraction changes in the Tienshan Mountains. Because of this, we focus here on determining snowfall proportions in precipitation by using combined multi-source data to analyze changes in the S/P and the impact of these changes on the snowmelt-dominated high-mountain regions of Central Asia.

## Results

Changes in either or both forms of precipitation (snowfall and rainfall) influence the S/P. Thus, to better understand how changes in S or P or both have influenced the S/P trend, this section provides a detailed description of long-term spatial variations of S, P and the S/P, based on the newly constructed data series for the Tienshan Mountains, Central Asia, during 1960–2017. The impacts of temperature on daily and seasonal changes of the S/P are also examined.

### Relationship of precipitation and snowfall to S/P

**Figure 2:**
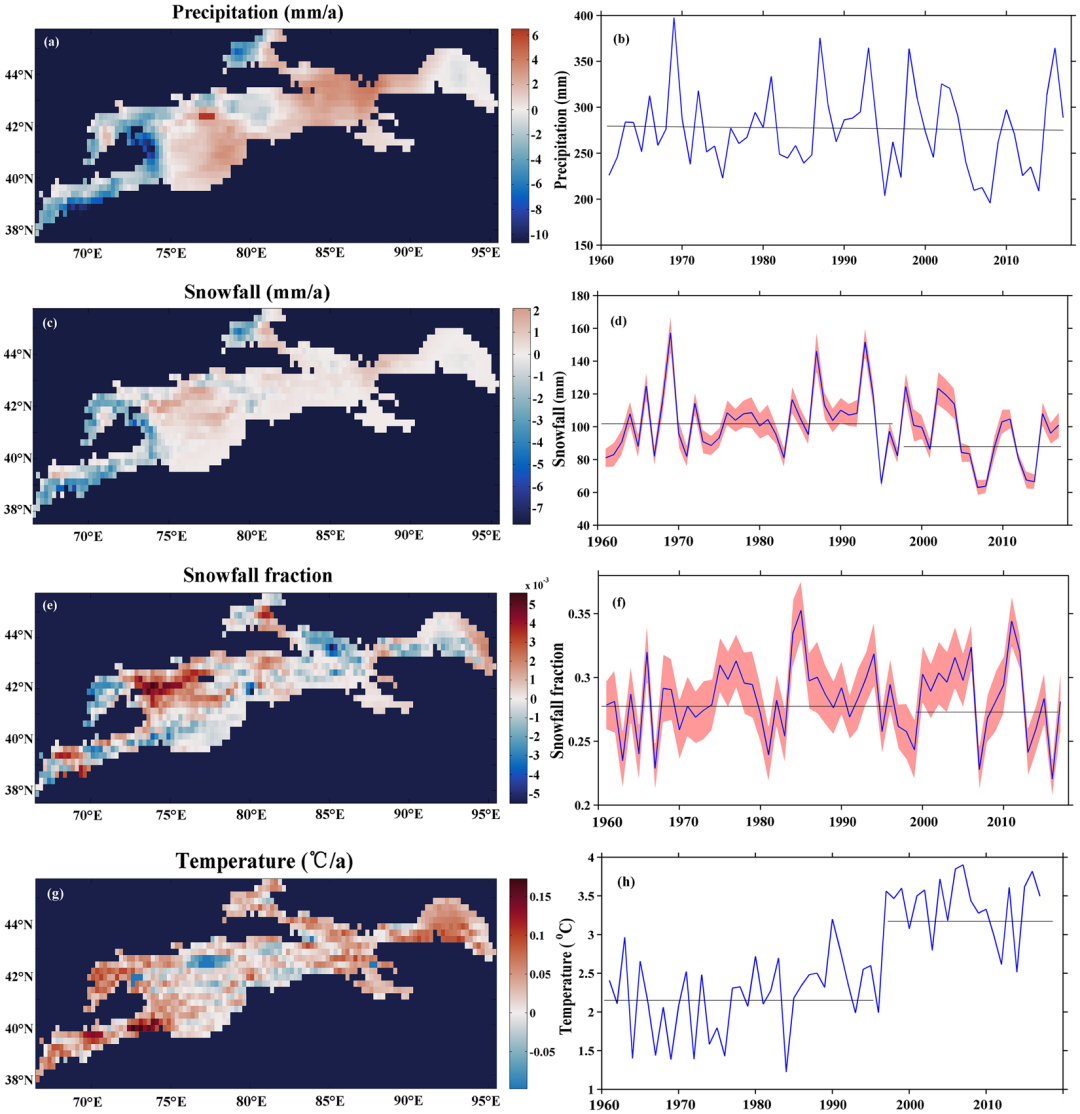
(**a**) Spatial tendency variations of precipitation over 1960–2017, (**b**) variations in precipitation over time, (**c**) spatial tendency variations of snowfall, (**d**) variations in snowfall over time (we partitioned snow from rain using temperatures between −1 °Cand 2 °C, the shaded pink portion showed snowfall measured result every per 0.5 °Cbetween −1 °Cand 2 °C), (**e**) spatial tendency variations of S/P, (**f**) variations in S/P, (**g**) spatial tendency variations of temperature, and (**h**) variations in temperature (Generated by Matlab R2013a, URL: http://cn.mathworks.com/products/matlab/).

Spatial and temporal variations in precipitation and snowfall in the Tienshan Mountains are plotted in Fig. 2, showing the average annual precipitation being around 280 mm. Although the annual precipitation has increased only slightly during the past half-century (Fig. 2b), there were clear increasing trends in the northern parts of the Tienshan Mountains, but decreasing trends in the Amudarya River basin in the westernmost parts of the mountains (Fig. 2a). Unlike the continuously increasing precipitation trend globally^23,24^, precipitation in the Tienshans showed a slightly downward trend from 1998.

Figure 2c shows that the variations in snowfall almost coincided with precipitation but were not completely consistent. The snowfall series can be divided into two periods, with the jump point occurring around 1993. During the first period (1960–1993), snowfall fluctuated with an increased rate of 0.46 mm/year, while during the second period (1994–2017), there was a reverse in the decrease at a rate of 1.34 mm/year (Fig. 2d). Spatially, the greatest increase in precipitation occurred in the north Tienshan Mountains, while the greatest increase in snowfall occurred in the northern slope of the western Tienshan Mountains (Fig. 2c). Overall, there were similar reductions in both snowfall and precipitation in the westernmost parts of the Tienshan Mountains.

The S/P was strongly influenced by variations in snowfall and precipitation, and temperature is a crucial factor affecting the precipitation form. Despite relatively uniform precipitation and snowfall trends, the S/P showed significant spatial differences. Figure 2f charts the variations in the S/P. As can be seen, the S/P series has been divided into two periods: before and after 1998. The pre-1998 series fluctuated at an increased rate of 0.6%/decade, while the post-1998 series fluctuated at a rate of −0.7%/decade (Fig. 2f). The S/P experienced a slight downward trend as a whole in the Tienshan Mountains, and can be divided into two periods of decline following the post-1998 increase. About 50% of the S/P grid boxes experienced negative trends. The areas with decreasing trend magnitudes are situated in the westernmost southern slope of the central, north and western part of east Tienshan Mountains (Fig. 2e). The decrease in the S/P suggests that changes to this measurement indicator are mainly related to changes in snowfall (with the correlation coefficient R = 0.4), with only a slight effect resulting from changes in precipitation (with the correlation coefficient R = 0.1). Decreases in snowfall coincided with consistent decreases in S/P, but increases in snowfall have been associated with either decreases or increases in S/P.

### Temperature-induced decreases in S/P at mid-low elevations

**Figure 3:**
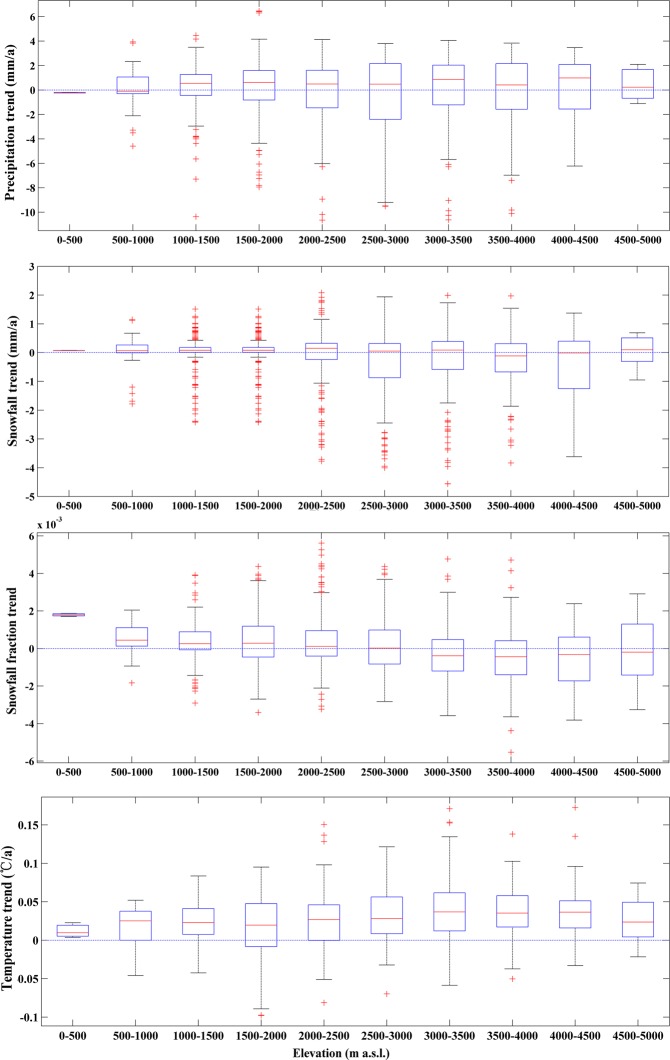
Variations in precipitation, snowfall, snowfall fraction and temperature across different elevations (On each box, the central mark indicates the median, and the bottom and top edges of the box indicate the 25th and 75th percentiles, respectively. The whiskers extend to the most extreme data points not considered outliers, and the outliers are plotted individually using the ‘+’ symbol) (Generated by Matlab R2013a, URL: http://cn.mathworks.com/products/matlab/).

Variations in snowfall amounts were the main contributors to changes in the S/P, but temperature is a crucial factor affecting snowfall^25^. Nearly all the regions in the Tienshan Mountains experienced obvious warming in the past half-century (Fig. 2g). The temperature rising trend began to accelerate from the late 1970s, with each decade warmer than the previous one, and a sharp increasing trend was experienced around 1998 (Fig. 2h). Although the average temperature in the Tienshan Mountains showed a rising trend, there still existed spatial difference. The most obvious warming areas were the westernmost portions of west, north and east Tienshan Mountains, while the eastern portion of west Tienshan Mountains (the Issyk Kol river basin) underwent a slight temperature reduction. Air temperature generally declines with increases in elevation, so the relationship between the S/P and elevation should be considered when analyzing any changes in S/P. Figure 2c,g indicate that low temperature is associated with high S/P, whereas high temperature is associated with low S/P. It is worth noting, however, that the rise in temperature did not lead to a consistent decrease in the S/P. Almost all elevations experienced a continuous warming trend, but the trend noticeably slowed for elevations over 3500 m (Fig. 3).

Boxplot creates box plots of the distribution in precipitation, snowfall, snowfall fraction and temperature across different elevations. Precipitation and snowfall changes for various elevations showed no significant differences. Nearly 20% of the Tienshan Mountains is situated below 1500 m, and around 74% of the S/P grids in this lower elevation region experienced a slight increasing trend. At middle elevations ranging between 1500 and 3500 m, there was a decrease in the S/P in about 53% of the grids. However, in altitudes above 3500 m (which accounts for about 16% of the Tienshan Mountains area), there were very small decreases in the S/P, with about 32% of the grids showing an increasing trend due to the temperature always remaining below freezing. In this region, precipitation mostly occurs in the form of snowfall due to the weakening negative effects of temperature change. Besides air temperature, other meteorological factors could also affect the S/P, e.g. increased saturation vapor pressure due to the rising temperature boosted evaporation and sublimation^26^ (Fig. 3).

### Precipitation shifts in snowfall and rainfall in the cold and warm seasons

The present study focuses on changes in snowfall and rainfall proportions in both the cold and warm seasons (the cold season being November-April and the warm season being May-October). The proportions are based on the percentage of snowfall or rainfall in the cold or warm seasons in relation to precipitation for the entire year.

**Figure 4:**
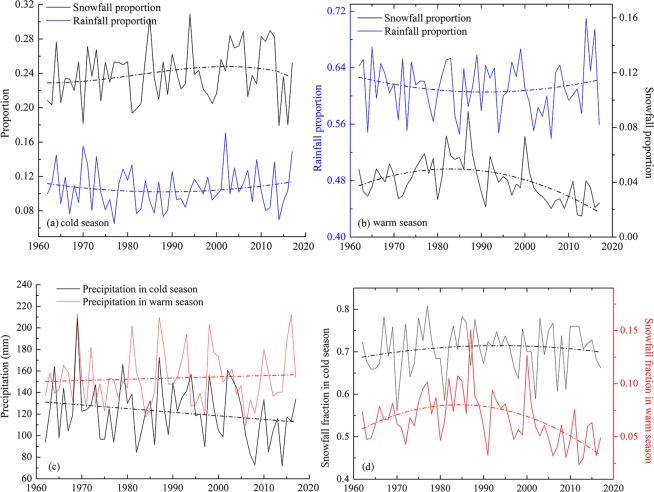
(**a**) Average values of snowfall and rainfall proportions in the cold season in the whole study area; (**b**) snowfall and rainfall proportions in the warm season; (**c**) precipitation changes in cold and warm seasons, respectively; and (**d**) snowfall fraction changes in cold and warm seasons, respectively (Generated by Matlab R2013a, URL: http://cn.mathworks.com/products/matlab/).

As can be seen in the Fig. 4, in 1960–2017, precipitation increased in the warm season but decreased in the cold season (Fig. 4c). However, the snowfall proportion decreased in the cold season following the sudden jump decreased in warming around 1998, while the rainfall proportion increased in the cold season (Fig. 4a). A similar phenomenon occurred in the warm season (Fig. 4b) around the mid-1980s. Regarding seasonal changes in the S/P, Fig. 4d shows decreases in both the cold and warm seasons. Totally speaking, the cold season exhibited a decrease in precipitation, especially snowfall, whereas the warm season showed an increase in precipitation dominated by rainfall.

### S/P changes associated with mountainous streamflow and water resources

The decrease in the S/P following the rising temperature could be quite important because such changes might influence the timing of the spring runoff of snow/glacier feed rivers and cause water shortages in summer^13^.

**Figure 5:**
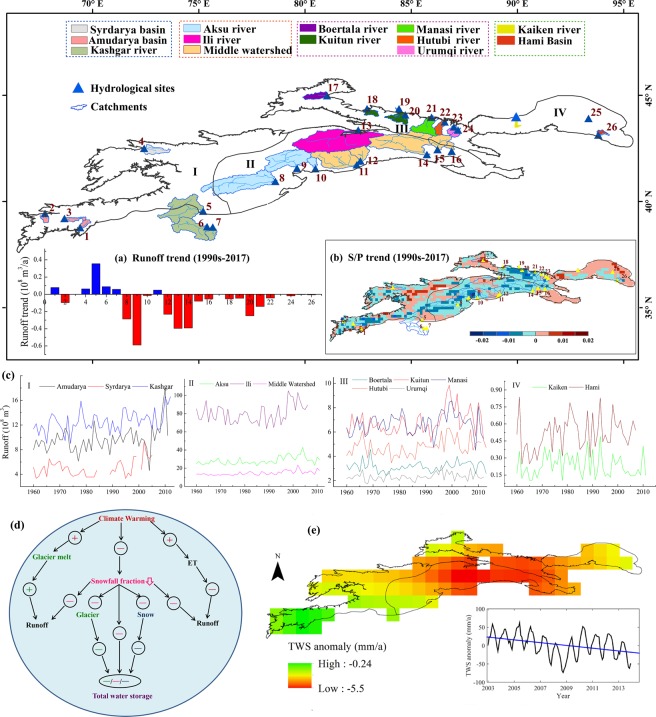
(**a**) Runoff variations after 1990s. (**b**) S/P trend in the same period. (**c**) Runoff variations for typical river basins in the (I) West Tienshan Mountains, (II) Middle Tienshan Mountains, (III) North Tienshan Mountains, and (IV) East Tienshan Mountains. The location of hydrological stations used in the study: 1. Dagana; 2. Sudzhina; 3. Takfon; 4. Karaoy; 5. Karabaly; 6. Kelak; 7. Shaman; 8. Shaliguilank; 9. Xiehela; 10. Tailan; 11. Heizi; 12. Heizi Reservoir; 13. Kafuqihai; 14. Dashankou; 15. Huangshuigou; 16. Kerguty; 17. Wenquan; 18. Jingheshankou; 19. Jilede; 20. Kenswat; 21. Jiangjunmiao; 22. Shimen; 23. Zhicaichang; 24. Yingxiongqiao; 25. Erdaogou; and 26. Toudaogou. (**d**) Schematic diagram of the potential impacts of climate change on water resources. (**e**) Changes in total water storage (Generated by ArcGIS 10.3, URL: http://www.esri.com/software/arcgis/arcgis-for-desktop, and Matlab R2013a, URL: http://cn.mathworks.com/products/matlab/).

There are numerous criss-crossing transboundary rivers shared by several countries in the Tienshan Mountains region of Central Asia. The variations in runoff in the largest rivers since 1960 are charted in Fig. 5c. In general, runoffs show increasing trends. Unlike the hydrologic stations in 4–7 of Kashgar river showed continuous increasing trend, other runoffs showed somewhat decrease after mid 1990s along with the jump in warming and an obvious declining trend in the S/P since the 1990s (Fig. 5a,b). By applying the Budyko water balance framework, Berghuijs *et al*.^6^ placed the observed long-term streamflow and precipitation measurements in the context of the Budyko hypothesis, and found that decreases in annual S/P are almost always associated with decreases in annual streamflow. For instance, in the Amudarya basin in the westernmost Tienshan Mountains, stations 1, 2 and 3 showed decrease in the S/P as well as decreased streamflow trend, whereas stations 4–7 showed an increase in the S/P and an increasing streamflow trend. Meanwhile, the southern part of the middle Tienshan Mountains (II) and east Tienshan Mountains (IV) exhibited a somewhat consistent change between the S/P and streamflow.

Recent studies on runoff trends of catchments that originate from the Tienshan Mountains indicate complex responses to climate change^15^. It appears that catchments which have a higher fraction of glacier/snow-dominated areas that feature a high S/P showed mainly increasing runoff trends in the past, while catchments with less or no glacierization and snow exhibited large variations in the runoff changes (IV part). As well, more uncertainties are expected in streamflow prediction in the precipitation and glacier/snow melt recharged rivers.

Warming is expected to significantly affect accelerated glacier and snow shrinkage and a decrease in snowfall fraction. Seasonal snow cover and glaciers are expected to change their water storage capacity under the ongoing warming climate in the mountainous region. The total water storage (TWS) indicates a decreasing trend based on GRACE data in the Tienshan Mountains in 2003–2013, with a decline rate of −3.72 mm/year (Fig. 5e), especially in the Middle Tienshan Mountains, falling at a rate of −5.5 mm/year^15^.

Figure 5d showed the schematic diagram of the potential impacts of climate change on water resources. Over the last half century, Tienshan Mountains in Central Asia has experienced obvious warming, the temperature experienced a sharp increase in 1998. The increases in temperature have outpaced precipitation. These changes have already impacted water resources significantly. Accelerating glacier melt driven by rising temperature caused an increase in runoff that benefited water resources. But the increase in evapotranspiraion (ET) driven by rising temperature caused the decrease in soil moisture and runoff, and then produced a negative impact on water resources. A decreased fraction of precipitation as snowfall was associated with lower accumulation of glaciers and snow, which produced a negative impact on total water storage. The predicted ongoing warming and reduction in snowfall fraction will inevitably influence the accumulation and melting processes of snow and glaciers, which will then further influence the streamflow and terrestrial total water storage.

## Discussions

### Determining precipitation form in mountains

Currently, the main methods used to discriminate (i.e., determine) snowfall proportions in precipitation are the temperature threshold methods, such as double critical temperature, single critical temperature, and temperature with relative humidity. Based on a thousand surface meteorological observations obtained from meteorological stations, Auer^27^ concluded that all precipitation falls as snow when the temperature is below 0 °C and that all precipitation falls as rain when the temperature is above 6.1 °C. Berghuijs *et al*.^6^, however, took 1 °C as the temperature threshold, while Dai^28^, based on the recorded data f 15,000 terrestrial meteorological stations from 1977–2007, held that precipitation phase-transition occurs in temperatures ranging from about −2 °C~4 °C over low-elevation land and −3 °C~6 °C over the ocean. Other studies adopted temperature thresholds ranging between 0 °C and 2 °C^29^.

We used the best currently available data, but this information is still not perfect. A detailed comparison of the data and method validation for the S/P calculations showed that changes in the S/P over time have been extremely difficult to determine accurately. Despite showing large year-to-year variations, the data is still unclear as to whether there have actually been long-lasting changes. Sun *et al*.^30^ emphasize the importance of fully accounting for natural variability when assessing long-term precipitation change.

The present study sets the precipitation partition between snow and rain at −1 °C~2 °C in the Tienshan Mountains. This temperature range was chosen because, under windy conditions, the catch difficulty of snowfall is larger than that of rainfall^10,31,32^, which can lead to overestimations of snowfall under conditions that mixed snow and rain occur. The change in precipitation from snow to rain can cause important and complex alterations in mountainous runoff processes, which is why this topic has been a focus of study for many scholars.

### The S/P change trends and its influencing factors

Given the ongoing high volatility in a warming climate, the S/P, along with the amount, frequency, and form of precipitation, will likewise undergo fluctuations^33,34^. Knowles *et al*.^11^ suggested that daily minimum temperature significantly affects snowfall to rainfall changes, and that strong snowfall decreased when wet-day minimum temperatures exceeded −5 °C. Feng and Hu^10^ indicated that the S/P has greatly decreased in the central United States and Pacific Northwest, but that the magnitude of the decrease has been lower in the eastern United States. Nonetheless, the S/P has shown a significant decreasing trend across the New England states, which is related to the North Atlantic Oscillation and the Pacific-North America index^9^. Similar decreasing trends in snowfall are found in Switzerland^5^, the continental United States^10,11^, the Tibetan Plateau^8,12^, and the Chinese Tienshan Mountains^3^. Krasting *et al*.^35^ indicated that annual snowfall is projected to decrease across much of the Northern Hemisphere during the 21^st^ century under the RCP4.5 scenarios, but that increases are projected for higher latitudes.

Increases in greenhouse gas emissions are causing sudden sharp warming and increased precipitation in the mid-high latitudes of the Northern Hemisphere^1^. Screen and Simmonds^25^ showed that the S/P is mainly related to changes in snowfall and that there are many factors influencing these changes. For instance, in the continental United States, the S/P is not only affected by the long period warming, but also by the Pacific Decadal Oscillation^11^. In the Swiss Alps, the northern mountains are mainly affected by temperature, whereas the southern mountains are impacted by the North Atlantic Oscillation^36^. More attention should be paid to how large-scale climate change (e.g. circulation and water vapor sources) causes changes in the S/P.

### Influence of S/P changes on watershed hydrological processes

Streamflow and precipitation are the two key observables of long-term planning for water resources, agriculture, and irrigation and for their associated infrastructure. The influence of rainfall or snowfall on streamflow cannot be simply considered. In the arid and semi-arid regions such as southeast Australia, during droughts, the reduced water availability leads to nonstationarity in rainfall- streamflow relationships^37^. Changes in the S/P cause subsequent changes in mean-annual and inter-annual streamflow, especially in rivers mainly supplied by glacier/snow melt water^38^. In the Alps river basin in northern Italy, research shows that decreases in the S/P may cause water shortages or flooding disasters in summer^39^. A decrease in the S/P elsewhere in the world could lead to future regional water shortages^40^. By applying a water balance model based on the Budyko framework to catchments located throughout the United States, Berghuijs *et al*.^6^ demonstrated that a higher fraction of precipitation falling as snow is associated with higher mean streamflow, but further studies are required to understand and respond appropriately to the consequences.

In the Tienshan Mountains, the topographic complexity and climate differences caused by wide ranges in elevation lead to significant spatio-temporal differences in how rain-snow-glacier melting water affects streamflow^41^. Decreases in the S/P will cause a delay in the start of the snow season as well as an early end to it, and will also affect the accumulation and melting processes of glaciers. Thus, changes in streamflow and water resources will be much more complex, affected by changes in rain-snow-glacier components. For a longterm continuous warming, high-Mountains in Central Asia may experience a risk shift in its hydrological cycle from one dominated by cold season snowfall and warm season glacial melt transfer to dominated by summertime rainfall, more uncertainties are expected in runoff prediction in the glacier/snow melt and precipitation recharged rivers. Therefore, clarifying the influence mechanism of S/P changes, the response of the S/P to climate change, and the sensitivity of streamflow to S/P changes will lay a scientific basis for water resources management in Central Asia and the Silk Road Economic Belt construction. More attention needs to be paid to the uncertain influences of large-scale climate change (e.g., circulation) on changes in the S/P.

## Methods

### Integration and continuation of precipitation datasets

**Figure 6:**
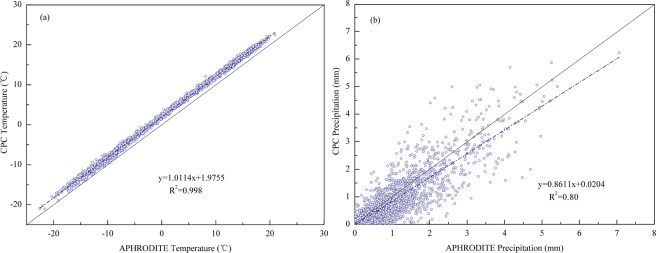
Regression correlation between APHRODITE and CPC daily temperature and precipitation data during 2000–2007; (**a**) is temperature, and (**b**) is precipitation (Generated by Matlab R2013a, URL: http://cn.mathworks.com/products/matlab/).

We selected daily temperature and precipitation data from APHRODITE and CPC for the overlapping period of 2000–2007, and plotted the data’s regression correlation Figures. There were very close relationships between the APHRODITE and CPC data, especially for temperature, which showed a coefficient of determination of R^2^ = 0.998 (Fig. 6a). The coefficient of determination of precipitation reached R^2^ = 0.80. (Fig. 6b).

The research used a per-pixel unary linear regression model in the overlap period to integrate different data sources to obtain new data series from 1960–2017.

### Validation of data and method

**Figure 7:**
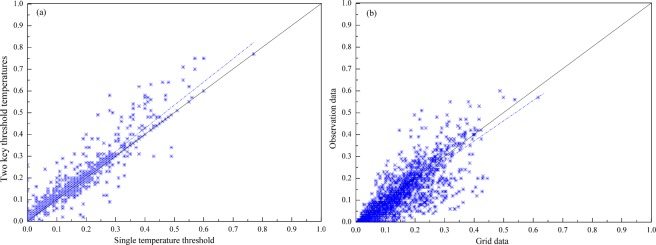
(**a**) Comparison of two temperature thresholds based on the 27 observational stations in the Tienshan Mountains (China part), the X-axis is S/P based on a single threshold and Y-axis is S/P based on two key threshold temperatures in each station from 1960–2017, (**b**) Comparison of S/P from observational data and corresponding grid data based on the single temperature threshold method (Generated by Matlab R2013a, URL: http://cn.mathworks.com/products/matlab/).

Many scholars used two methods of single temperature threshold and two key threshold temperatures^6,8^ to separate the rain and snow based on a daily average temperature. In order to compare the results of the two methods of single temperature threshold and two key threshold temperatures, we used the two methods to estimate S/P respectively based on the 27 observational stations data (daily air temperature, precipitation, relative humidity, and pressure) (Fig. S1). The results showed that the correlation coefficient of the two methods is reach to R^2^ = 0.92 (Fig. 7a).

We used the 27 stations’ observational data and extract the corresponding grid data from 1961–2017. Based on the single temperature threshold method, we compared the estimated S/P results of grid data and observational data. The results showed that the correlation coefficient is R^2^ = 0.61 (Fig. 7b). From Fig. 7 we can see that the data and method we used are suitable for the S/P estimation in the Tienshan Mountains, Central Asia.

### Single temperature threshold

Compared with other datasets, the new data series this paper used are more accurate, thus making them the most suitable resource for research on the Tienshan Mountains. The limitation of these gridded data is the lack of relative humidity (RH) data. Therefore, we selected the single temperature threshold to separate snowfall and rainfall. Precipitation which occurs on days with an average temperature below the temperature threshold is considered to be entirely snowfall, whereas precipitation which occurs on days with temperature above the temperature threshold is considered to be entirely rainfall.

In the Tienshan Mountains, with their complex topography and large differences in elevation, determining the temperature threshold may take different elevations into consideration. In the 27 meteorological stations located in the mountains, snowfall and rainfall are recorded separately. Figure S2 plots the rain and snow data for different temperatures. Twenty of the stations are situated at low elevations (below 1500 m), 5 are situated at intermediate elevations (between 1500 m and 3000 m), and 2 are situated at relative high elevations (above 3000 m). The records for the 27 stations provide weather codes from 1960 to 1979: snow (including sleet and snowstorms) is marked as 31XXX (XXX being the actual amount), and a mixture of rain and snow is marked as 30XXX. Daily mean temperatures for the two types are selected, and then temperature ranges for the two are acquired for each station, from which the critical temperatures for snow and rain are determined. We partitioned snow from rain using temperatures between −1 °Cand 2 °C. By taking the estimation error of different temperature thresholds into consideration, we were able to discriminate snowfall from daily precipitation at every 0.5 °C point between −1 °C and 2 °C.

### Two key threshold temperatures

The fraction of precipitation falling as snow is approximated using a temperature threshold for each day of recorded data^7,27,42^. Temperature threshold methods use temperature or wet-bulb temperature and relative humidity to define thresholds which demarcate precipitation types (Eqs. 1–8).

1 $${P}_{{\rm{type}}}=\{\begin{array}{c}snow,\,{T}_{W}\le {T}_{min}\\ sleet,\,{T}_{min}\le {T}_{W}\\ rain,\,{T}_{W}\ge {T}_{max}\end{array}\le {T}_{max}$$

where P_type_ is the daily precipitation types (snow, rain, or sleet), T_w_ is daily wet-bulb temperature, and T_min_ and T_max_ are threshold temperatures.

Daily wet-bulb temperature (T_w_) contains air temperature, humidity, and pressure information and can be calculated by the following:

2 $${T}_{w}={T}_{a}-\frac{{e}_{sat}({T}_{a})\times (1-RH)}{0.000643\times {P}_{s}+\frac{\partial {e}_{sat}}{\partial T}}$$

3 $${e}_{sat}({T}_{a})=6.1078\times {e}^{(\frac{17.27\times {T}_{a}}{{T}_{a}+237.3})}$$

where T_a_ is daily temperature, P_s_ is daily air pressure (hPa), and e_sat_ (T_a_) is the saturation vapor pressure (hPa).

T_max_ is the temperature value at which the occurrence probabilities of rain and sleet are equal to each other, and T_min_ is that for the equal occurrence probabilities of snow and sleet. T_max_ and T_min_ were calculated by the following equations:

4 $${T}_{min}=\{\begin{array}{c}{T}_{0}-\Delta S\times ln[{e}^{(\frac{\Delta T}{\Delta S})}-2{e}^{(-\frac{\Delta T}{\Delta S})}],\,\frac{\Delta T}{\Delta S} > ln2\\ {T}_{0},\,\frac{\Delta T}{\Delta S}\le ln2\end{array}$$

5 $${T}_{max}=\{\begin{array}{c}2\times {T}_{0}-{T}_{min},\,\frac{\Delta T}{\Delta S} > ln2\\ {T}_{0},\,\frac{\Delta T}{\Delta S}\le ln2\end{array}$$

where ΔT and Δ S are the temperature difference and temperature scale, respectively.

6 $$\Delta {\rm{T}}=0.215-0.099\times {\rm{RH}}+1.018\times R{H}^{2}$$

7 $$\Delta {\rm{S}}=2.374-1.634\times {\rm{RH}}$$

where RH is daily relative humidity of each observational meteorological stations.

T_0_ depends on both elevation and RH and is given by the following:

8 $${T}_{0}=-5.87-0.1042\times Z+0.0885\times {Z}^{2}+16.06\times RH-9.614\times R{H}^{2}$$

### Data analysis

27 meteorological stations in the Tienshan Mountains were collected from the Meteorological Administration of China (http://data.cma.cn/site/index.html), which have complete records of almost all the climatic factors from 1958-present, including daily observations of temperature (minimum, maximum and average) at 2 m height, wind speed measured at 10 m height, precipitation, relative humidity, sunshine duration, and average vapour pressure, etc (Table S1). The daily precipitation are recorded as snowfall and rainfall separately before 1979, and then were recorded uniformly as precipitation. Of these 27 stations, 5 were below the altitude of 500 m, 6 were at 500–1000 m, 9 were at 1000–1500 m, 3 were at 1500–2000 m, 3 were at 2500–3000 m, and 1 station were above 3500 m. To ensure the quality (i.e. credibility and homogeneity) of the data, datasets that were missing more than 5% or missed more than 5 days in a month were removed from the study.

In the most alpine regions or regions with harsh climate, there were limited number of gauging stations, some scholars have chosen reliable grid data or reanalysis data to estimate S/P. In order to offset the limitation of the sparsity of observational stations in the high mountain regions of Central Asia, we used the datasets on daily temperature and precipitation from the Asian Precipitation-Highly Resolved Observational Data Integration Towards Evaluation (APHRODITE) (http://www.chikyu.ac.jp/precip/english/products.html) and the National Oceanic and Atmospheric Administration (NOAA) Climate Prediction Center (CPC) (https://www.esrl.noaa.gov/psd/data/gridded/data.cpc.globalprecip.html). The APHRODITE datasets were generated from the rain gauge observational networks in Asia. Compared with other datasets, the APHRODITE are more accurate, as they develop long-term daily grid precipitation and temperature data from rain-gauge observation records across Asia provided by international collaborations with local meteorological/hydrological agencies and researchers. APHRODITE, are high quality datasets, given their genesis in international collaboration and quality control, thus making them the most suitable resource for research on the Tienshan Mountains in Central Asia^43–46^. The dataset resolution is 0.25° × 0.25° and the duration is 1951 to 2007. To extend the duration, we also used CPC daily data from 1979-present, at a resolution of 0.50° × 0.50°. This dataset is part of products suite from the CPC Unified Precipitation Project. The primary goal of the project is to create a suite of unified precipitation products with improved quality and consistent quantity by combining all information sources available at CPC and taking advantage of the optimal interpolation (OI) objective analysis technique. Many scholars have used the dataset globally and regionally^47–49^.

Monthly surface runoff data from 1960–2014 on river basins that originated from the Tienshan Mountains (e.g., Aksu River, Ili River, Middle watershed, Boertala River, Kuitun River, Manasi River, Hutubi River, Urumqi River, Kaiken River and Hami Basin) were taken from the flow-control stations out of the mountains for each river, obtained from the Regional Hydrological Bureau in China. Annual surface runoff data for rivers outside China (Syr Darya Basin, Amu Darya Basin, Issky-Kol Basin) were obtained from the Global Runoff Data Centre (https://www.bafg.de/GRDC/EN/Home/homepage_node.html), local hydrology bureau, and relevant references^50^. It is worth noting that many hydrological monitoring stations and routine surveillance programs ceased to operate following the collapse of the Soviet Union in the early 1990’s.

## Supplementary information

Supporting-Information.
