# Supplementary material for: Declining snowfall fraction in the alpine regions, Central Asia
Source: Sci Rep. 2020 Feb 26;10:3476. doi: 10.1038/s41598-020-60303-z (PMC7044428; doi:10.1038/s41598-020-60303-z)
Supplement: Supplementary file 1 — Supporting-Information. [file 41598_2020_60303_MOESM1_ESM.pdf]

Supporting Information for

Declining snowfall fraction in the alpine regions, Central Asia

Zhi Li[1,2], Yaning Chen[1,2*], Yupeng Li[1,2], Yang Wang[3]

[1] State Key Laboratory of Desert and Oasis Ecology, Xinjiang Institute of Ecology and Geography, Chinese Academy of Sciences, Urumqi 830011, China

[2] University of Chinese Academy of Sciences, Beijing, 100049, China

[3] College of Pratacultural and Environmental Sciences, Xinjiang Agricultural University, Urumqi 830052

Contents of this file

Table S1
Figures S1 to S2

Table S1: Observational Stations:

Stations	ID	Elevation (m)	Longitude	Latitude
Aheqi	51711	1985	78.45	40.93
Aksu	51628	1104	80.23	41.17
Alashankou	51232	285	81.05	40.50
Alaer	51730	1012	82.58	45.18
Balikun	52101	1637	93.00	43.60
Baicheng	51633	1229	84.15	43.03
Bayanbulak	51542	2458	81.90	41.78
Caijiahu	51365	441	87.53	44.20
Dabancheng	51477	1104	88.32	43.35
Hami	52203	737	93.52	42.82
Jinghe	51334	320	82.90	44.62
Kashi	51709	1387	75.59	39.28
Keping	51720	1162	79.05	40.50
Kuche	51644	1099	82.95	41.72
Korla	51656	932	86.13	41.75
Luntai	51642	976	88.22	42.23
Qitai	51379	794	84.25	41.78
Torugart	51701	3504	91.63	43.48
Turpan	51573	35	89.57	44.02
Wenquan	51330	1355	75.40	40.52
Urumqi	51463	918	89.20	42.93
Ulugqat	51705	2176	81.02	44.97
Usu	51346	479	87.62	43.78
Yanqi	51567	1056	75.25	39.72
Yining	51431	663	84.67	44.43
Yiwu	52118	2908	86.57	42.08
Zhaosu	51437	1851	81.33	43.95

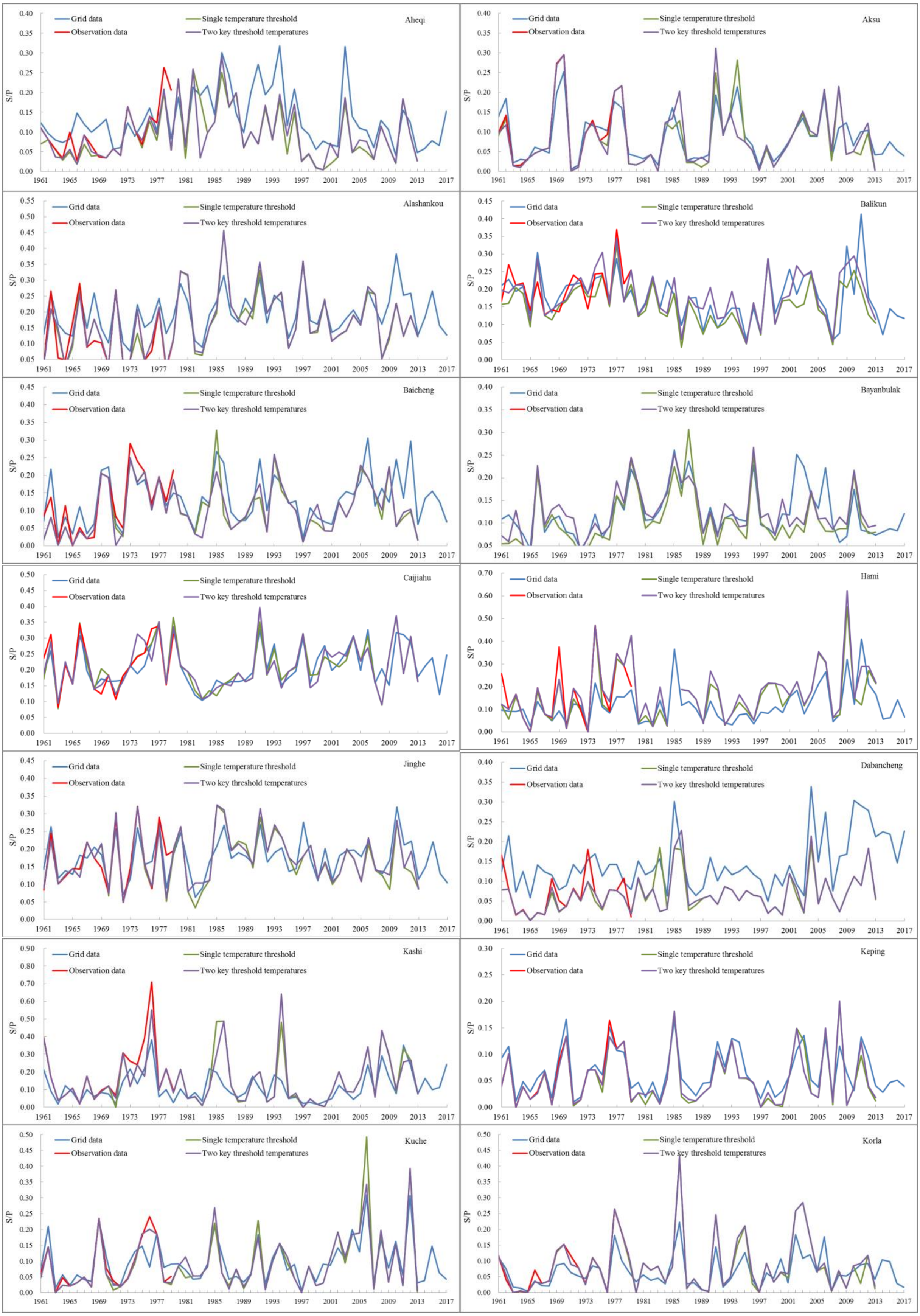

Grid data
Observation data
Single temperature threshold
Two key threshold temperatures
S/P
Aheqi
Aksu
Alashankou
Balikun
Baicheng
Bayanbulak
Caijiahu
Hami
Jinghe
Dabancheng
Kashi
Keping
Kuche
Korla
1961
1965
1969
1973
1977
1981
1985
1989
1993
1997
2001
2005
2009
2013
2017

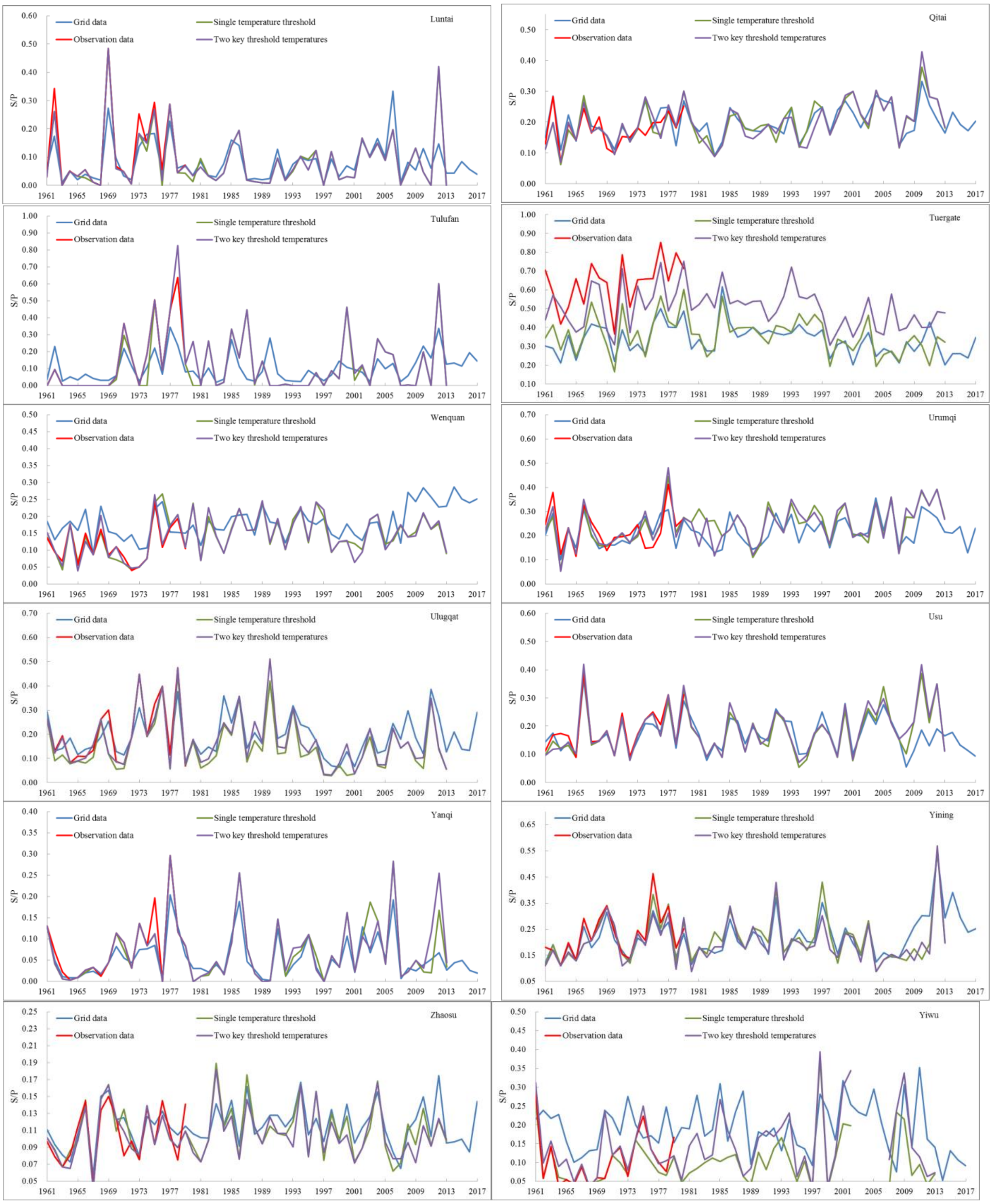

Fig. S1 The snowfall fraction (S/P) results from different methods over 1960-2017 and observational data from 1960-1979: **Blue line (Grid data):** S/P was estimated by the single temperature threshold based on the grid data that corresponding to each observation station. **Red line (Observation data)**: The records for the 27 stations provide weather codes from 1960 to 1979: snow (including sleet and snowstorms) is marked as 31XXX (XXX being the actual amount). **Green line (Single temperature threshold)**: S/P was estimated by the single temperature threshold based on the data of each observation station. **Purple line (Two key threshold temperatures)**: S/P was estimated by the two key threshold temperatures based on the data of each observation station.

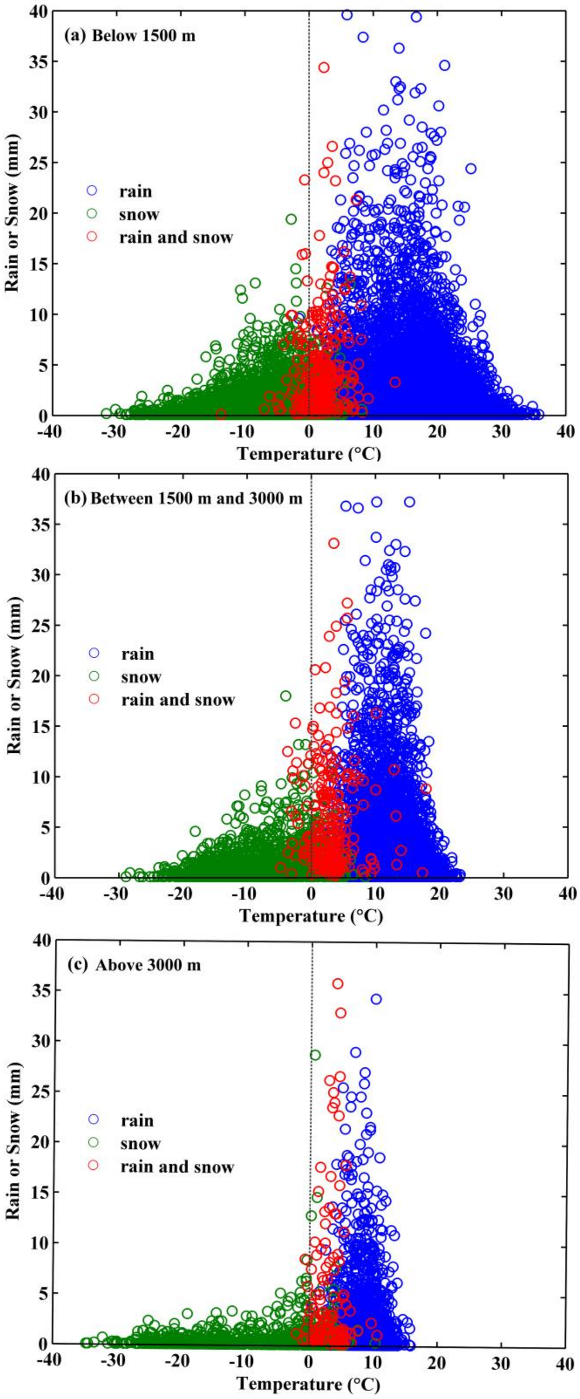

Fig. S2 Plot of precipitation type (rain vs snow) for different air temperature bands, based on all stations located in the Tienshan Mountains (China part) from1960 to 2017; a) 20 stations were located at low elevations (below 1500 m), b) 5 stations were located at intermediate elevations (between 1500 m and 3000 m), and c) 2 stations were located at high elevations (above 3000 m).
